# Video analysis of patients with blepharospasm and lower face dystonias

**DOI:** 10.3389/dyst.2023.11385

**Published:** 2023-06-15

**Authors:** Mahdieh Hosseini, Panagiotis Kassavetis, Mark Hallett

**Affiliations:** 1 National Institute of Neurological Disorders and Stroke (NINDS), Human Motor Control Section, National Institutes of Health, Bethesda, MD, United States; 2 Adult Neurology Residency Program, University of California, Los Angeles, Los Angeles, CA, United States; 3 Neurology Department, University of Utah, Salt Lake City, UT, United States

**Keywords:** time, severity, sensory trick, blepharospasm, scale

## Abstract

**Background:**

Blepharospasm (BSP) is a focal dystonia. There is a lack of standardization in the length of time necessary to get a measure of BSP severity for rating scales.

**Objectives:**

1) Determine the difference between evaluating the number of eye closures in patients with blepharospasm in 1 vs. 2 min. 2) Characterize the prevalence, phenomenology and concordance of sensory trick in subjects with only blepharospasm compared to those with blepharospasm associated with other dystonias of the head.

**Methods:**

Thirty-eight, 2-min-long standardized videos of subjects with BSP without any other dystonias were reviewed (group1). Eye closure rate was measured in 0–60 s vs. 60–120 s. Wilcoxon signed-rank test and Spearman correlation coefficient were used to compare the eye closure rate between these two intervals. An additional 68 standardized videos of subjects with blepharospasm associated with dystonia of the head were reviewed (group2). Presence, phenomenology and concordance between what subjects verbally reported as their sensory trick and what they demonstrated was classified for both groups then qualitatively compared.

**Results/conclusion:**

Eye closure rates between 0–60 s and 0–120 s were not statistically different. There is no added benefit of counting the number of eye closures in 2 min, compared to 1 min, in patients with BSP. Sensory trick was reported by 57% of subjects with BSP and 80% of subjects who have blepharospasm and other dystonias of the head. With 100% and 97% concordance, patients’ self-reported sensory trick accurately describes the movements that alleviate their dystonic movements.

## Introduction

Blepharospasm (BSP) is a type of focal dystonia, characterized by involuntary stereotyped, bilateral, and synchronous contractions of the orbicularis oculi muscles, leading to partial or total eyelid rim closure [1]. Precise clinical rating scales of severity are important to assess a patient’s condition and the outcome after treatment. However, there is a lack of standardization in length of time necessary to get a reliable measure of BSP severity. The Blepharospasm Severity Rating Scale (BSRS), which is the most specific scale for BSP, was validated based on segments of video recordings that were 120 s long [2]. The Burke-Fahn-Marsden (BFM) dystonia scale was initially validated with videos of 4 min and 15 s [3]. Later the BFM scale, and the Global Dystonia Rating Scale (GDRS), were validated with videos that used the Dystonia Study Group Videotape Examination Protocol, which records the eyes while open and at rest for 30 s [4]. The latest version of this protocol (Version 40) includes a 1-min-long segment at rest with eyes open, and 2 min in a BSP sub-group in the “Blepharospasm Tools” protocol [5]. It is not clear if 2 min of patient observation is more reliable than 1 min in estimating the severity of BSP symptoms. In this study we aim to determine if there is a difference between evaluating the number of eye closures in patients with blepharospasm in 1 vs. 2 min in an effort to guide clinicians rating severity of blepharospasm.

Contraction of the orbicularis oculi muscle can be brief or prolonged, relieved or aggravated by certain actions. Tricks (or alleviating maneuvers) are defined as episodic and specific maneuvers that alleviate dystonic movements [6]. They may be pure sensory such as a light stroke on the face. Sensory tricks can also be motor, where they involve a motor task such as chewing. Subjects may also utilize forcible tricks where they mechanically hold the dystonic body part to resist the involuntary contraction. Finally, patients can have mixed tricks that involve a combination of sensory, forcible, and/ or motor tricks. Sensory tricks can play a role in the quality of life of patients with dystonias [7]. There are few studies that characterize sensory trick in patients with blepharospasm [8], and even fewer that study it in comparison to other focal dystonias [9]. The Dystonia Coalition video database offers an opportunity to study the prevalence and phenomenology of sensory trick in subjects with only blepharospasm in comparison those who have blepharospasm in conjunction with other dystonias of the head including lower face, larynx, jaw, and tongue. Sometimes in neurology clinical practice, what patients self-report, such as tremor, is not in concordance with what is found on physical examination. Video recordings offer an opportunity to retrospectively explore the congruence of what subjects verbalize as their sensory trick compared to what can be demonstrated, here termed as concordance.

## Methods

**FIGURE 1:**
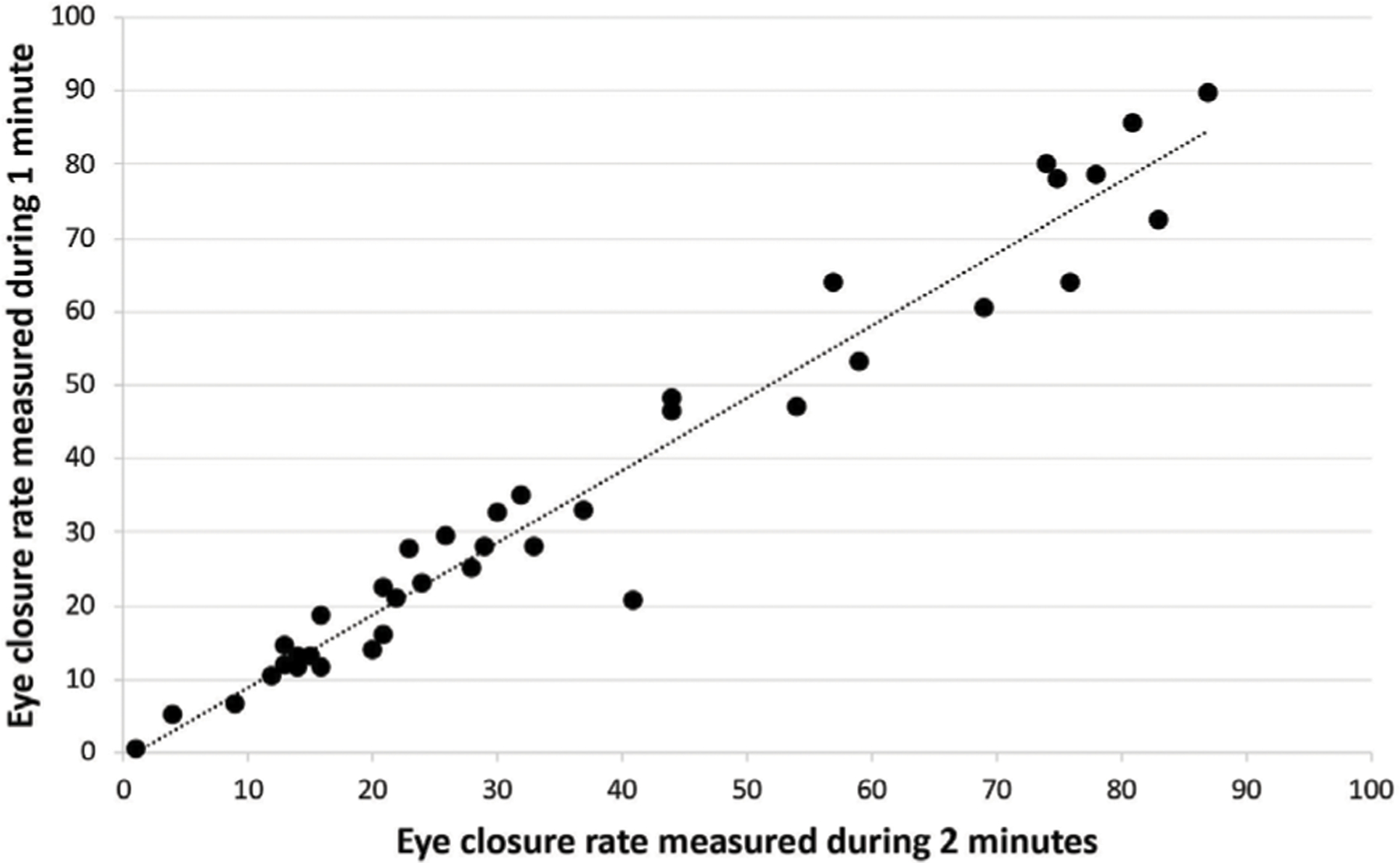
Bivariate correlation of the eye closure rate between one vs. 2 min in patients with blepharospasm.

In this study we used data from the Dystonia Coalition (DC) video database. The Dystonia Coalition is a large consortium of investigators, a collaboration of medical researchers and patient advocacy groups to advance clinical and translational research in the dystonias [10]. The DC is supported by the Office of Rare Diseases Research (ORDR) in the National Center for Advancing Translational Sciences (NCATS) and The National Institute of Neurological Disorders and Stroke (NINDS) at the NIH [10]. For the first objective, video recordings of all subjects with pure blepharospasm from the Dystonia Coalition video database were reviewed. Of them, 38 had blepharospasm without any other dystonias. This classification was verified by the authors. Thus 38, 2-min-long standardized videos were included in the analysis. Eye closure was defined as a new and full contact between borders of upper and lower lids, without making a distinction between blinks, spasms, and apraxias of eyelid opening. The number of eye closures were counted by a first reviewer, in the intervals 0–60 s and 60–120 s for each subject. These counts were verified by a second reviewer. The speed of videos was slowed as needed down to ×0.5 for subjects with high blink rates. The data were nonparametric, therefore non-parametric statistical tests were used for analysis. The eye closure rate per minute, was compared between 1 min (number of closures 0–60 s) and 2 min (number of closures 0–120 s/2) with Wilcoxon signed-rank test. Spearman correlation coefficient was used for bivariate correlation of the eye closure rate between the two intervals (Figure 1).

To achieve the second objective, two groups were studied. For the first group, the identical set of 38 videos of subject with pure BPS were included. Of these 38 subjects, thirty of them were asked about sensory trick on camera. For the second group, an additional 68 standardized videos of subjects with BSP associated with dystonia of the head, including lower face, larynx, jaw, and tongue were reviewed from the Dystonia Coalition video database. Of these 68, 59 of them were asked about the presence and demonstration of sensory trick on camera. Subjects in the second cohort can be further subdivided into “lower face” or “neck” dystonias. Lower face can be further categorized as tongue, jaw, or larynx. Of note, in this second group researchers ask “what maneuvers do you do to help alleviate abnormal movements?” without specifying maneuvers that uniquely help BSP.

For each subject the following were evaluated: 1) Does the subject self-report any maneuvers that alleviate abnormal (dystonic) movements? 2) The subject is then asked to demonstrate the verbalized sensory trick on camera. The concordance between the self-reported sensory trick and what was demonstrated on camera was determined. 3) The demonstrated trick was classified as pure sensory, forcible, motor trick, or a mix.

## Results

The patients with only blepharospasm had a range of severity. Their BFM eye score, as reported by the Dystonia Coalition, was 5.1 (SD 2.1, their range 0–8, with a maximum possible range of 0–16 for this severity score). Their GDRS upper-face score was 4.4 (SD 2.1, their range 1–10, with a maximum possible range of 0–10 for this severity score). The mean age of these patients was 63.4 years, SD = 8.8, 19 females. The average age of onset and duration of illness in the two cohorts was similar. The mean age of subjects with BSP and lower face dystonias was 64.8 years, SD = 8.4. The duration of illness in each cohort was 13.8 years, SD = 12, and 11.6 years, SD = 10.5, respectively.

For an *α* = 0.05, sample size of 38, test statistic of 291, and critical value of 235, the Wilcoxon Signed Rank Test failed to reject the null hypothesis that there is no difference between eye closure rate in one vs. 2 min in patients with blepharospasm. We found that eye closure rates between 0–60 s and 0–120 s were not statistically different (*p* = 0.25) and showed significant positive correlation (*p* < 0.001).

Sensory trick was reported by 17/30 (57%) of subjects with BSP. The average total BFM severity score in this group was 5.1, SD = 2.1. There was total concordance between the reported and demonstrated trick in subjects who have BSP only (100%). Six subjects had pure sensory trick, nine had a forcible trick, and one had a mixed sensory and forcible trick. Two subjects reported that a task alleviated their blepharospasm. The most common trick was forcibly keeping the eyes open using index finger.

Subjects in the second cohort were further subdivided into either “lower face” or “neck” dystonias. Lower face dystonias were further categorized as tongue, jaw, or larynx (Table 1).

In comparison sensory trick was present in 47/59 (80%) of subjects who have blepharospasm associated with other dystonias of the head with 97% concordance (Table 2). The single subject with discordance reported their dystonic movements improved “when looking away and when at rest.” While “rest” is difficult to quantify on videotaping, the subject does not look away and continued to fidget when asked to demonstrate their sensory trick. The average BFM severity score (total score) in this group was 11.3 ± 4.3, notably higher than blepharospasm only group. In this group, 16 had pure sensory, 25 had forced, and eight had an action trick. Similar to the first group, the most common trick in this group was to forcibly holding either the upper or lower lid open using fingers.

## Discussion

In this study we used the Dystonia Coalition video database to examine the length of time necessary to count eye closures to get an accurate measure of blepharospasm severity. We also studied sensory tricks in subjects with blepharospasm in comparison to those with blepharospasm accompanied with other dystonias of the head. Our results show that there is no added benefit of counting the number of eye closures in 2 min, compared to 1 min, in patients with BSP. In blepharospasm literature, various studies explore the effect of botulinum toxin on blink rate [11]. Fewer studies explore the effect of light, eating, conversation on blink rate in blepharospasm [12]. However, there are no studies that address the time necessary to examine these eye closures in patients with blepharospasm to get a reliable measure of severity in various circumstances. The current version of the Dystonia Coalition video protocol, which employs 1 min, can be used for assessment of BSP. We propose that for the development of video-database protocols of patients with blepharospasm in the future, video recordings of 1 min are just as good as 2 min to assess severity. A limitation of this study is that a distinction was not made between blinks, spasms, and apraxias of eyelid opening. The results here cannot necessarily be applied if it is desired to separate different types of eye closures or if the evaluation is done in a situation different from rest.

In this study we find that subjects who have blepharospasm associated with other dystonias of the head (80%) are more likely to utilize and rely on sensory tricks compared to subjects who have only blepharospasm (57%). This ratio of sensory trick is much closer to the Benign Essential Blepharospasm Research Foundation’s 2021 survey, which reports a 77% prevalence of sensory trick [13]. In an observational study, Pandey et al. [9] analyzed the frequency, types, phenomenology, and effectiveness of sensory tricks in patients suffering from primary blepharospasm or idiopathic cervical dystonia. They reported presence of sensory trick in 18 out of 20 patients with blepharospasm (90%). In comparison they find that sensory trick is present in 7 out of 20 subjects with cervical dystonia without blepharospasm (35%). This trend contrasts our findings, though both of our comparison groups included BSP in their diagnosis.

We found an interesting resemblance in distribution of the type of trick in the two groups we studied groups (Table 2). In both groups 53% of subjects relied on a pure forced sensory trick. The most common type of trick was mechanically holding the upper and/or lower lid open with dominant fingers. Subjects who had BSP with other dystonias of the head were more likely to utilize mixed tricks (5% vs. 28%).

Apart from one subject in the second group, we found that there was near total concordance between what patients reported and demonstrated as their sensory trick. This suggests that self-reported sensory trick accurately describes the movements that alleviate dystonic movements. Researchers and clinicians caring for patients with blepharospasm can accurately rely on patients’ self-reported sensory trick. Finally, a limitation of this study is that the wording that examiners used to inquire about presence and demonstration of sensory trick is not specific to maneuvers that help BSP but may suggest all abnormal movements.

## Figures and Tables

**TABLE 1:** Distribution of lower face dystonias.

(4/59)–7% reported jaw dystonia (6/59)–10% reported laryngeal dystonia (confirmed by nasopharyngoscopy) (38/59)–64% reported neck dystonia Remaining 19% have unspecified lower face dystonias

**TABLE 2:** Prevalence and phenomenology of sensory trick in patients with BSP and those with BSP associated with other dystonias of the head.

	BSP only	BSP + lower head dystonia
Total present	17/30	47/59
Concordant	17/17	46/47
Not Concordant	0	1
Pure Sensory	6 (35%)	16 (34%)
Pure Forced	9 (53%)	25 (53%)
Action	2 (12%)	8 (17%)
Mixed	1 (5%)	13 (28%)

## Data Availability

The data analyzed in this study is subject to the following licenses/restrictions: belongs to Dystonia Coalition. Requests to access these datasets should be directed to MHa, hallettm@ninds.nih.gov.
